# Usefulness of cortisol/ACTH ratio (CAR) for diagnosis of cushing's syndrome: comparison of CAR with findings in dexamethasone suppression test

**DOI:** 10.1038/s41598-022-22676-1

**Published:** 2022-10-21

**Authors:** Yuichiro Iwamoto, Fuminori Tatsumi, Takashi Itoh, Taku Sasaki, Shigehito Mori, Toshitomo Sugisaki, Erina Nakao, Mana Ohnishi, Takashi Kusano, Haruka Takenouchi, Hideyuki Iwamoto, Junpei Sanada, Yoshiro Fushimi, Yukino Katakura, Tomohiko Kimura, Masashi Shimoda, Shuhei Nakanishi, Kohei Kaku, Tomoatsu Mune, Hideaki Kaneto

**Affiliations:** Department of Diabetes, Endocrinology and Metabolism, Kawasaki Medical School, 577 Matsushima, Kurashiki, 701-0192 Japan

**Keywords:** Endocrinology, Medical research

## Abstract

Cushing's syndrome and subclinical Cushing's syndrome (SCS) are conditions of increased cortisol secretion from the adrenal glands. Cushing's syndrome includes adrenocorticotropic hormone (ACTH)-dependent Cushing's syndrome (Cushing's disease) and ACTH-independent Cushing's syndrome (AICS). The purpose of this study was to investigate the diagnostic potential of the cortisol / adrenocorticotropic hormone (ACTH) ratio (CAR) for diagnosis of Cushing's syndrome or SCS in adult subjects. This was a single-center, retrospective, observational study. This study enrolled 44 subjects with SCS, 14 AICS, 10 CD, and 248 non-Cushing's syndrome subjects who had undergone a 1 mg dexamethasone suppression test (1 mg DST). Definition of SCS was as follows: no physical signs characteristic of Cushing syndrome and cortisol was ≥ 83 nmol/L in 1 mg DST. The diagnostic potential of CAR for diagnosis of Cushing's syndrome was evaluated by comparing the correlation between CAR and after-load cortisol level in 1 mg DST. As the results, there was a strong positive correlation between CAR and after-load cortisol level in subjects with AICS (*r* = 0.800, *p* < 0.005). CAR was 10,040 ± 4170 nmol/pmol in subjects with NCS, 17,535 ± 10,246 nmol/pmol in SCS, 101,221 ± 18,009 nmol/pmol in AICS, and 4324 ± 2051 nmol/pmol in CD, all of which were significantly higher compared to those with AICS (*p* < 0.0005). The cutoff values of CAR for screening at our institution were 11,849.6 nmol/pmol for AICS (AUC 0.935, *p* < 0.005, sensitivity 92.3%, specificity 83.5%) and 7006.1 nmol/pmol for CD (AUC 0.714, *p* < 0.05, sensitivity 100.0%, specificity 46.8%). There was a positive correlation between CAR and adrenal adenoma diameter in subjects with AICS (*r* = 0.508, *p* < 0.05), but there was no correlation between tumor diameter and CAR in subjects with SCS and CD. In conclusion, high CAR indicates increased cortisol secretion from the adrenal glands. Since CAR is a simple indicator that can be easily evaluated by general practitioners as well as endocrinologists, we think CAR would be useful for the early detection of Cushing's syndrome.

## Introduction

Cushing's syndrome is a chronic excess production of cortisol from the adrenal glands and is broadly classified into adrenocorticotropic hormone (ACTH)-independent Cushing's syndrome (so-called adrenocorticotropic Cushing's syndrome) and ACTH-dependent Cushing's syndrome (so-called Cushing's disease)^1^. Subclinical Cushing's syndrome (SCS) is not accompanied by the characteristic physical signs of Cushing's syndrome^2^ but shows autonomous cortisol secretion. Subjects with SCS may also be at risk of developing into Cushing's syndrome. Cushing's syndrome, including subclinical one, causes hypertension, and glucose intolerance, and cardiovascular events and finally leads to shorter healthy life expectancy and life expectancy^3–5^.

The 1 mg dexamethasone suppression test (1 mg DST) is a test to evaluate cortisol autocrine secretion from the adrenal glands by measuring early morning cortisol levels after taking 1 mg dexamethasone at 23:00 on the previous day, and when the after-load cortisol level is 3 μg/dL or higher, cortisol autocrine secretion is considered to be enhanced^6,7^. It is very important to confirm cortisol autocrine secretion, because it has been reported that the mortality is increased at after-load cortisol levels of 3 µg/dL or higher in 1 mg DST^8^. However, it is sometimes difficult to perform 1 mg DST in non-specialized institutions or in ambulatory care settings. If a method for diagnosis of Cushing’s syndrome could be established only with basic endocrine hormone levels without performing 1 mg DST, treatment could be initiated more easily and at an earlier stage. Primary aldosteronism is another relatively common adrenal disease and brings about secondary hypertension. For diagnosis of the disease, aldosterone level and renin activity are measured, and aldosterone / renin activity ratio is very often utilized as established diagnostic criteria for primary aldosteronism in clinical practice^9,10^. Similarly, in diagnosis of Cushing’ syndrome, cortisol and ACTH levels are very often measured. However, cortisol/ ACTH ratio (CAR) has not been established yet as clear diagnostic criteria for Cushing’ syndrome. In this study, we investigated whether CAR would be useful for the diagnosis of Cushing's syndrome in subjects who underwent 1 mg DST by directly comparing CAR with findings in 1 mg DST.

## Material and methods

### Study subjects

Subjects eligible for the present study were patients who visited the endocrinology outpatient clinic in Kawasaki School Hospital between January 1st, 2010 and March 31st, 2021. The study protocol including the opt-out informed consent was approved by Institutional Review Board of Kawasaki Medical School (No. 5531–00). The study was conducted in accordance with the Declaration of Helsinki. Since this study was retrospective, instead of obtaining informed consent from each patient, we provided public information about the study via the hospital homepage.

A total of 327 subjects who underwent 1 mg DST between January 1, 2010 and March 31, 2021 were selected as this study participants; subjects younger than 20 years old, pregnant, and taking corticosteroids were excluded from the study (3 cases). Subjects with diseases that could affect ACTH and cortisol secretion were excluded (1 case with Addison's disease, 1 case with anorexia nervosa, 1 case with hypoproduction of ACTH, 2 cases with growth hormone-secreting tumors, 1 case with congenital adrenocortical enzyme deficiency, and 1 case with ACTH unresponsiveness). Participants included 248 subjects with non-Cushing’s syndrome (NCS), 44 with subclinical Cushing’s syndrome (SCS), 14 with ACTH independent Cushing’s syndrome (AICS), and 10 with Cushing's disease (CD).

### Methods

Analysis was performed using age, height, body weight, BMI, systolic and diastolic blood pressure, and blood test results at the time when 1 mg DST was performed. The data were collected from medical records. All cases were diagnosed with SCS, AICS or CD after 1 mg DST was performed. Adrenal adenoma diameter was evaluated by abdominal CT in 35 cases with SCS and 14 cases with AICS. Pituitary tumor diameter was evaluated by brain MRI in 9 cases with CD.

### Adrenocortical hormone measurements

ACTH and cortisol blood samples were taken upon awakening after 20–30-min fasting. Safter serum separation on ice cooling, ACTH was measured by ECLIA method using cobas e 801 (Roche Diagnostics K.K, Switzerland). Cortisol was measured by CLIA method using the ADVIA Centaur XP (Siemens Healthcare Corp., Tokyo). CAR was calculated by the following formula: CAR = (plasma cortisol (mol/L)/(plasma ACTH (pool/L))*100. For the 1 mg DST procedure, 1 mg dexamethasone was taken at 23:00 on the day before the test day, and cortisol was measured at 8:00.

### Diagnosis of Cushing's syndrome

Cases of exogenous glucocorticoid excess were excluded in this study. SCS was diagnosed when there were no physical signs characteristic of Cushing syndrome and cortisol level was ≥ 83 nmol/L in 1 mg DST. AICS was diagnosed when cortisol level was ≥ 1 pmol/L in 1 mg DST and ACTH level was suppressed. CD was diagnosed when findings included loss of cortisol diurnal variation, late night cortisol ≥ 207 μg/dL, and increased urinary free cortisol. CD was assessed by 0.5 mg DST, 8 mg DST, CRH load test, brain MRI, and cavernous sinus sampling from pituitary tumor. The diagnosis was made when there was ACTH autonomous secretion.

### Statistical analysis

This was a cross-sectional study of patients who underwent 1 mg DST. The primary endpoint was the diagnostic value of CAR for diagnosis of subclinical Cushing's syndrome and Cushing's syndrome, and patients were divided into four groups: NCS, SCS, AICS, and CD. The Shapiro–wilk test was used to evaluate the normal distribution of both CAR and natural logarithmized post-load cortisol, the Kuruskal Wallis test was used for a 4-group comparison, and the Steel–Dwass test was used for a post hoc test. Pearson product-moment correlation coefficient was used to evaluate the correlation between CAR and cortisol level and the correlation between tumor diameter and each parameter. ROC curves were developed to determine the cutoff values for CAR and 1 mg DST in our facility. JMP (16.0.1) was used for the analysis and EXCEL for Mac (16.58) was used to create the figures.

## Results

### Clinical characteristics

**Figure 1:**
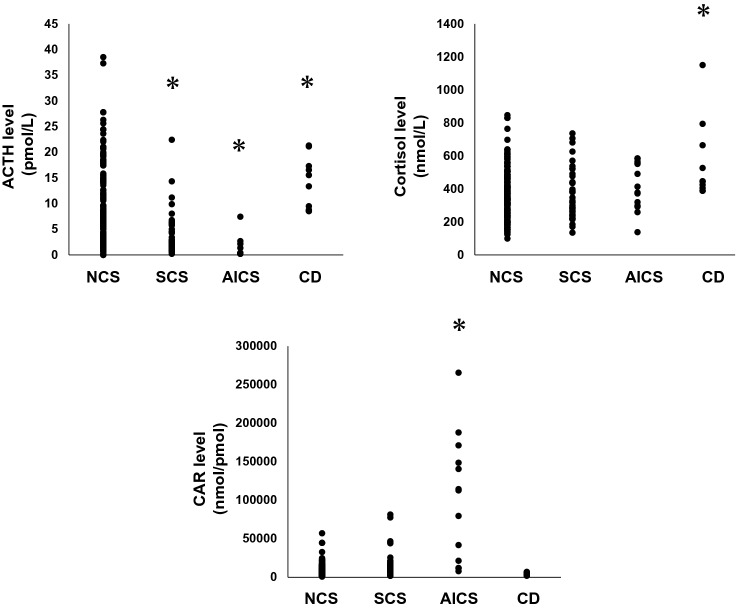
We plotted the distribution of ACTH, cortisol, and CAR in the NCS, SCS, AICS, and CD groups. *P < 0.05, Steel–Dwass test compared to NCS.

The clinical characteristics of the subjects in this study are shown in Table 1, and Fig. 1. The subjects with SCS tended to be older compared to those in other groups. AICS and CD showed a greater proportion of women. BMI was significantly lower in CD group. Systolic and diastolic blood pressure did not differ among the groups. Early morning ACTH level was 7.4 ± 0.8 pg/mL in NCS group. ACTH level in subjects with SCS and AICS was 4.1 ± 1.9 pmol/L and 1.3 ± 3.4 pg/mL, respectively, both of which were significantly lower compared to those with NCS. ACTH level in subjects with CD was 14.1 ± 4.0 pg/mL, which was significantly higher than NCS group (*p* < 0.05). After-load cortisol level in 1 mg DST was 33.0 ± 8.3 nmol/L in NCS group. On the other hand, after-load cortisol level in subjects with SCS, AICS and CD were 102.7 ± 20.0 nmol/L, 387.0 ± 34.8 nmol/L and 248.9 ± 41.2 nmol/L, respectively. All were significantly higher than NCS group (*p* < 0.0005). The same analysis was performed for CAR. The CAR in AICS group was 101,221 ± 18,009 nmol/pmol, which was significantly higher than that in NCS group (10,040 ± 4170 nmol/pmol) (*p* < 0.0005). The CAR in SCS and CD group was not different from that in NCS group.

**Table 1:** Comparison of various values among participants in this study with non-Cushing's syndrome (NCS), subclinical Cushing's syndrome (SCS), ACTH-independent Cushing’s syndrome (AICS) and Cushing's disease (CD).

Parameter	All subjects (*n* = 316)	NCS (*n* = 248)	SCS (*n* = 44)	AICS (*n* = 14)	CD (*n* = 10)
Male/female	159/157	129/119	22/22	3/11	5/5
Age (years)	58.1 ± 15.0	57.5 ± 14.7	65.1 ± 12.6*	52.7 ± 17.7	50.8 ± 18.9
Body weight (kg)	67.0 ± 18.3	68.1 ± 18.2	65.2 ± 19.0	59.7 ± 14.3*	58.2 ± 18.8
BMI (kg/m^2^)	25.7 ± 6.1	25.7 ± 5.8	24.5 ± 4.2	24.5 ± 4.2	22.9 ± 5.6
Systolic blood pressure (mmHg)	138.8 ± 20.6	138.6 ± 21.1	138.1 ± 17.9	142.0 ± 18.3	141.4 ± 20.4
Diastolic blood pressure (mmHg)	81.2 ± 15.4	81.2 ± 15.9	79.6 ± 12.4	83.2 ± 17.4	83.8 ± 12.5
Blood glucose (mg/dL)	127.3 ± 42.5	129.2 ± 43.6	120.1 ± 31.6	121.0 ± 48.5	97.5 ± 13.7
HbA1c (NGSP,%)	7.24 ± 2.57	7.22 ± 2.36	7.23 ± 3.38	7.58 ± 3.99	7.68 ± 3.28
Serum sodium (mmol/L)	139.5 ± 8.8	139.5 ± 9.3	139.5 ± 6.0	141.1 ± 2.3	139.5 ± 3.8
Serum potassium (mmol/L)	4.37 ± 6.01	4.45 ± 6.52	4.04 ± 0.57	3.64 ± 0.28	4.02 ± 0.43
Serum chloride (mmol/L)	103.8 ± 5.1	103.8 ± 5.0	103.3 ± 6.7	104.5 ± 2.6	102.3 ± 4.3
Plasma osmolality (mOsm/kg)	288.5 ± 7.1	288.8 ± 6.9	286.6 ± 9.1	286.9 ± 3.8	389.8 ± 4.7
Plasma ACTH (pmol/L)	6.9 ± 0.7	7.4 ± 0.8	4.1 ± 1.9*	1.3 ± 3.4*	14.1 ± 4.0*
Plasma cortisol (nmol/L)	373.4 ± 17.1	364.7 ± 19.2	365.3 ± 46.5	414.8 ± 78.6	563.7 ± 93.0*
Plasma DHEA-S (nmol/L)	3.0 ± 0.3	3.1 ± 0.3	1.9 ± 0.7*	1.9 ± 1.3	4.8 ± 1.4*
PRA (mcg/mL/hr)	1.57 ± 2.68	1.54 ± 2.62	1.65 ± 2.60	2.64 ± 4.65	1.13 ± 1.08
PAC (pmol/L)	3585 ± 429	3686 ± 468	3178 ± 1302	2623 ± 2565	2923 ± 2951
Plasma dopamine (pmol/L)	82.7 ± 31.1	86.7 ± 34.0	69.6 ± 98.4	40.0 ± 170.5	53.5 ± 215.7
Plasma noradrenaline (pmol/L)	1528.5 ± 125.0	1524.8 ± 135.5	1778.7 ± 352.2	922.9 ± 680.6	1443.5 ± 859.5
Plasma adrenaline (pmol/L)	135.6 ± 16.9	142.0 ± 18.5	117.1 ± 53.1	50.5 ± 92.1	103.7 ± 116.5
Urinary cortisol (nmol/24 h)	200.1 ± 30.3	173.1 ± 32.7	207.7 ± 83.5	420.4 ± 120.4*	407.7 ± 153.5*
Urinary aldosterone (nmol/24 h)	26.6 ± 4.0	27.5 ± 4.4	26.1 ± 12.6	19.8 ± 20.4	11.2 ± 24.8
CAR (nmol/pmol)	14,722 ± 4242	10,040 ± 4170	17,535 ± 10,246	101,221 ± 18,009*	4324 ± 2051
After-load ACTH in 1 mg DST (pmol/L)	0.8 ± 0.1	0.6 ± 0.2	0.5 ± 0.5	0.5 ± 0.6	7.5 ± 1.0*
After-load cortisol in 1 mg DST (nmol/L)	65.1 ± 11.6	33.0 ± 8.3	102.7 ± 20.0*	387.0 ± 34.8*	248.9 ± 41.2*

Data presented as mean ± standard deviation. *p < 0.05 with Steel–Dwass test compared to NCS. *BMI* Body mass index; *ACTH* Adrenocorticotropic hormone; *PRA* Plasma renin activity; *PAC* Plasma aldosterone concentration; *CAR* Cortisol / ACTH ratio; *1 mg DST* 1 mg dexamethasone suppression test.

### Relationship between CAR and after-load cortisol level in 1 mg DST

**Figure 2:**
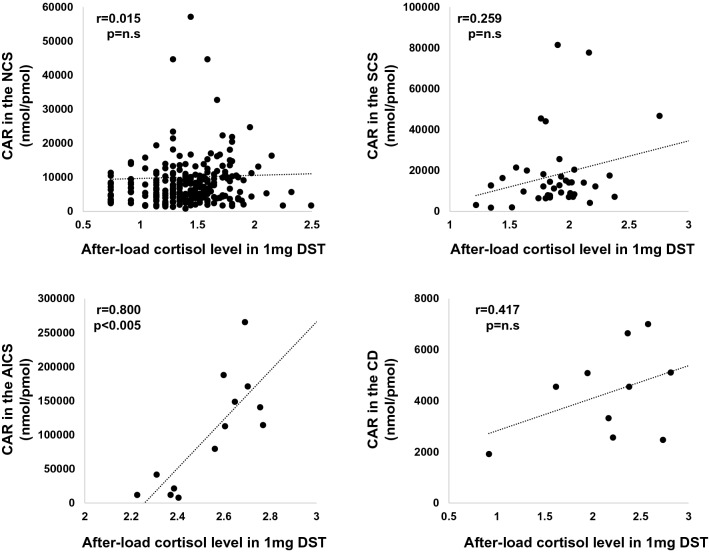
We evaluated the single correlation between CAR and after-load cortisol level in 1 mg DST in the NCS, SCS, AICS and CD group. There was a strong positive correlation between CAR and after-load cortisol level in AICS group, whereas there was no significant single correlation between them in NCS, SCS and CD group.

The correlation between CAR and after-load cortisol level in 1 mg DST is shown in Fig. 2. There was a strong correlation between CAR and after-load cortisol levels in subjects with AICS (*r* = 0.800, *p* < 0.005), although there was no significant correlation between them in those with SCS (*r* = 0.259, *p* > 0.05) and CD (*r* = 0.417, *p* > 0.05).

### Cutoff values for CAR and after-load cortisol level for diagnosis of SCS and Cushing’s syndrome

**Figure 3:**
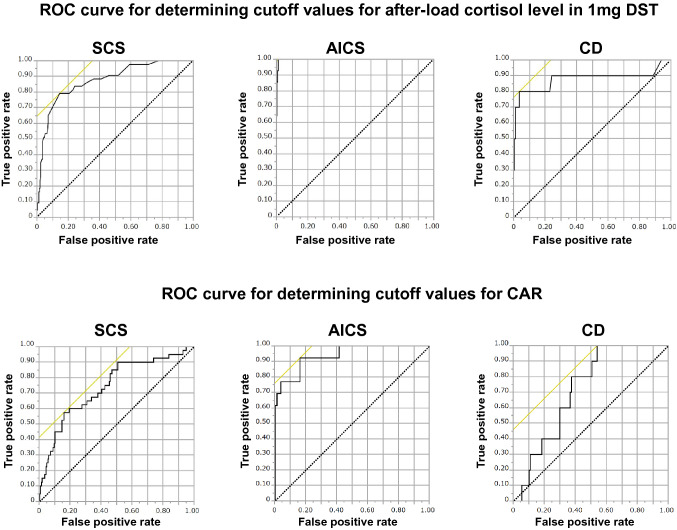
ROC curves were created for determining cutoff values for after-load cortisol level in 1 mg DST and cutoff values for CAR to diagnose Cushing's syndrome. The upper panel is the ROC curve for after-load cortisol level in 1 mg DST and the lower panel is the ROC curve for CAR.

ROC curves were created to obtain cutoff values for CAR and after-load cortisol level for the diagnosis of SCS and Cushing’s syndrome. Results are shown in Table 2 and Fig. 3. The cutoff value of after-load cortisol level for diagnosing SCS was 55.2 nmol/L (sensitivity 79.1%, specificity 83.7%, *p* < 0.005), and CAR was not useful for detecting SCS (*p* > 0.05). The cut-off value of post-load cortisol level to diagnose AICS was 168.3 nmol/L (sensitivity 100%, specificity 83.6%, *p* < 0.005). The cutoff value of CAR was 11,849.6 nmol/pmol (sensitivity 92.3%, specificity 83.5%, *p* < 0.005). The cut-off value of after-load cortisol level to diagnose CD was 88.3 nmol/L (sensitivity 80.0%, specificity 96.4%), and the cut-off value of CAR was 7006.1 nmol/pmol (sensitivity 100%, specificity 46.8%, *p* < 0.05).

**Table 2:** ROC curves to determine the cutoff value of the post-loading cortisol level in 1 mg DST and CAR for diagnosing each disease.

	AUC	*p* value	Cut-off	Sensitivity	Specificity
**After-load cortisol level in 1 mg DST**					
Subclinical Cushing’s syndrome	0.873	< 0.005	55.2	79.1	83.7
ACTH-independent Cushing’s syndrome	0.998	< 0.005	168.3	100.0	83.6
Cushing’s disease	0.878	< 0.005	88.3	80.0	96.4
**CAR**					
Subclinical Cushing’s syndrome	0.741	n.s	–	–	–
ACTH-independent Cushing’s syndrome	0.935	< 0.005	11,849.6	92.3	83.5
Cushing’s disease	0.714	< 0.05	7006.1	100.0	46.8

1 mg *DST* 1 mg dexamethasone suppression test; *CAR* Cortisol/ACTH ratio.

Taken together, it seemed that a CAR of 11,849.6 nmol/pmol or higher would be useful for screening of AICS with a high sensitivity. When the cutoff value was set at 79,599.3 nmol/pmol, the specificity of AICS detection was very high (99.6%). Therefore, it seemed that a CAR of 79,599.3 nmol/pmol would be useful for definitive diagnosis of AICS. In addition, when the CAR was less than 7006.1 nmol/pmol, the sensitivity of CD detection was very high (100%). Therefore, it seemed that a CAR of 7006.1 nmol/pmol would be useful for screening of CD.

### Correlation between tumor diameter and CAR

**Figure 4:**
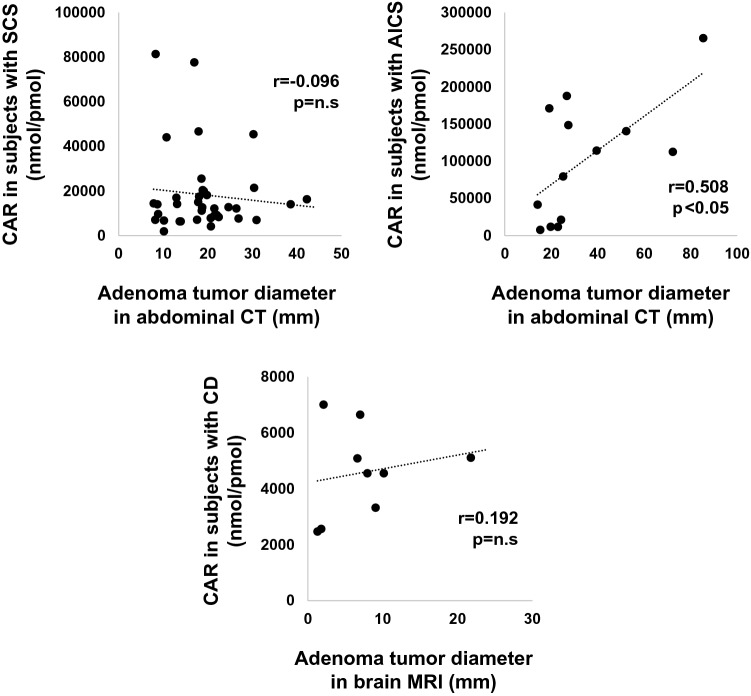
The single correlation between CAR and adenoma tumor diameter in each group was evaluated. For SCS and AICS, the largest adrenal adenoma diameter assessed by abdominal CT was used for evaluation. For CD, the maximum diameter of pituitary adenomas evaluated by brain MRI was used for evaluation. There was a positive correlation between adrenal adenoma diameter and CAR in subjects with AICS (*r* = 0.508, *p* < 0.05), whereas there was no correlation between CAR and tumor diameter in those with SCS and CD.

Next, we evaluated the possible correlation between CAR and maximum tumor diameter of adrenal adenomas in abdominal CT in subjects with SCS and AICS and pituitary adenomas in brain MRI in those with CD (Fig. 4). As the results, there was a positive correlation between adrenal adenoma diameter and CAR in subjects with AICS (*r* = 0.508, *p* < 0.05), although there was no correlation between CAR and maximum tumor diameter in subjects with SCS and CD.

## Discussion

After-load cortisol level in 1 mg DST is useful in detecting SCS and Cushing’s syndrome^7,10^. Although the ACTH / cortisol ratio has been reported to be useful in detecting Cushing's disease, it has not yet been established as diagnostic criteria in clinical practice^11^. In this study, there was a positive correlation between CAR and after-load cortisol level in 1 mg DST. There was also a positive correlation between adrenal adenoma diameter and CAR in subjects with AICS. We think that it is possible to diagnose and screening AICS when CAR is elevated, and screening CD when CAR is decreased.

As described above, it seems that CAR is useful for screening of CD, but we should be careful about the following point. As shown in Table 2, sensitivity of CAR for CD was high in this study. Therefore, we think that CAR could be a useful indicator in screening of CD when it is clearly decreased. In contrast, specificity of CAR for CD was quite low. Therefore, in cases of low CAR, load tests such as CRH load test and localization of ACTH-producing tumors should be performed for the diagnosis of CD. On the other hand, when the CAR was greater than 11,849.6 nmol/pmol, AICS was highly sensitive (92.8%), useful as a screening tool, and specificity was equivalent to that of 1 mg DST. A CAR of 79,599.3 nmol/pmol or greater was 99.6% specific, suggesting that AICS and CD may be useful as a definitive diagnostic tool. Since ACTH and cortisol are usually measured when Cushing's syndrome or adrenal disease is suspected, the CAR, which can be calculated by a simple formula, may be a useful indicator for estimating the presence of AICS clinically.

There are several limitations in this study. First, this is a single-center, retrospective, observational study. Although this study suggests that CAR has diagnostic potential for SCS and AICS, further studies with a larger number of cases are needed to generalize the results. Second, in this study, we failed to evaluate dexamethasone concentrations after 1 mg DST which made it difficult to precisely interpret the data in DST^12–14^. One reason for the low specificity of SCS and AICS at 1 mg DST in this study may be that the blood dexamethasone concentration was not sufficiently elevated. Finally, in this study, we were able to calculate test values for screening tests and definitive diagnosis when considering cutoff values with equivalent AUC in the ROC curve in AICS, but there were only 10 participants with CD, and the specificity did not change when the cutoff value was changed for CD. Further studies are needed on the usefulness of CAR in the practice of Cushing’s syndrome.

Multiple tests are required to diagnose Cushing's syndrome, including dexamethasone suppression testing, urinary cortisol storage, and imaging studies. Therefore, it is difficult for clinicians to diagnose the disease with a single test^15^. In addition, such diagnosis substantially depends on specialized endocrinologists who perform the loading tests and scintigraphy^16^. Therefore, if there is an indicator to strongly suspect AICS or CD using basic endocrine values, not only endocrinology specialists but also general practitioners would be able to easily differentiate AICS and CD. The data in this study suggest that CAR, which can be calculated only by endocrine tests at rest, may become a new index for detecting adrenogenic Cushing's syndrome and Cushing’s disease. We think that CAR would be promising for many clinicians to easily notice the possibility of Cushing’s syndrome at an earlier point and thus the findings in this study would be very informative and useful from the clinical point view.

## Data Availability

All data generated or analysed during this study are included in this published article.
